# Safety of videoconferencing for physical rehabilitation and exercise: A systematic review and meta-analysis

**DOI:** 10.1177/02692155251361916

**Published:** 2025-07-30

**Authors:** Riley CC Brown, Joshua Simmich, Robert Cuthbert, Megan H Ross, Pablo Molina-Garcia, Trevor G Russell

**Affiliations:** 1 RECOVER Injury Research Centre, 1974 The University of Queensland, Brisbane, Australia; 2 STARS Education and Research Alliance, Surgical Treatment and Rehabilitation Service, The University of Queensland and Metro North Health, Brisbane, Australia; 3 Instituto de Investigación Biosanitaria ibs.Granada, Granada, Spain

**Keywords:** Telehealth, telerehabilitation, adverse events, exercise, rehabilitation

## Abstract

**Objective:**

Investigate the safety of physical rehabilitation and/or exercise interventions conducted via videoconferencing.

**Design:**

Systematic review/meta-analysis.

**Data sources:**

PubMed, Web of Science, Embase and CINAHL from inception until 12 June 2025.

**Review methods:**

Trials including participants with chronic disease or history of restorative or reconstructive surgery implementing a physical rehabilitation or exercise intervention via videoconferencing compared to an in-person exercise comparator and reporting adverse events were included. Meta-analyses were conducted for between-group comparisons of adverse events using incidence rate ratios. Risk of bias was assessed using the Cochrane Risk of Bias 2 tool and the certainty of the evidence with Grading of Recommendations, Assessment, Development and Evaluation.

**Results:**

Out of 3436 records, 22 trials were included in this review (28 otherwise eligible trials were excluded for not reporting adverse events). No significant differences were observed between groups for minor/moderate [incidence rate ratio (IRR): 1.00, 95% CI: 0.71–1.41, *p* = 1.00] or major (IRR: 1.77, 95% CI: 0.55–5.70, *p* = 0.98) adverse events. Incidence was low for both videoconferencing (one every 410 sessions) and in-person (one every 414 sessions). Eighteen trials (82%) were graded ‘some concerns’ or ‘high’ on overall risk of bias score, primarily due to bias arising from measurement and selection of the reported adverse events. Certainty grading was ‘low’ for adverse event outcomes.

**Conclusion:**

This study suggests that there is no clear evidence of a difference in adverse event incidence between in-person and videoconferencing physical rehabilitation or exercise interventions. Future studies must improve transparency of defining and reporting to improve certainty in these findings.

## Introduction

In-person physical rehabilitation and exercise services for adults often have significant barriers that can influence participation. These barriers are complex and may include personal, logistical and healthcare system factors.^1^ Telehealth is a form of digital health and is defined as the remote provision of healthcare service using telecommunications technology over a distance.^2^ In physical rehabilitation and exercise settings, telehealth utilisation may lead to improved accessibility to care and support patient preferences.^3,4^ Videoconferencing interventions (using an audio-visual link) can often result in similar outcomes to in-person services.^4–6^

Clinician concern regarding the safety of videoconferencing services have been documented in previous literature,^7–9^ and is especially evident for high-risk and remote patient groups.^8,9^ A 2021 Australian report detailing allied health telehealth use identified that safety concerns relating to videoconferencing were present in 13% of surveyed clinicians.^7^ This observation was amplified for exercise physiologists, where 24% of clinicians reported safety concerns relating to the delivery of videoconferencing.^7^ However, only 4% of exercise physiologists reported experiencing an adverse event while conducting a videoconferencing session. Patient perceptions with videoconferencing were generally positive, with 80% of participants reporting high levels of perceived safety.^7^ Occurrence of adverse events during videoconferencing interventions are very low.^4,10^ Combined with the results of previous literature,^4,6^ these findings suggest that videoconferencing can be used as a safe medium for physical rehabilitation and exercise service delivery.

Previous literature investigating the safety of telehealth for a broad range of healthcare services concludes that it is not inferior to usual care in-person services for safety measures.^11^ However, there remains a need for a robust investigation into the safety of videoconferencing. Therefore, the objective of this systematic review was to compare the rate of minor/moderate and major adverse events of videoconferencing physical rehabilitation and exercise interventions compared to in-person services.

## Methods

This systematic review and meta-analysis was conducted and is reported according to the Preferred Reporting Items for Systematic Reviews and Meta-Analysis statement.^12^ This review was registered under the trial number CRD42024567928 through the PROSPERO International prospective register of systematic reviews on 31 July 2024.

### Terminology

Videoconferencing: The use of telecommunication in the form of an appointment over a two-way audiovisual platform.^13^ Videoconferencing interventions involve a health professional directly supervising participants performing physical rehabilitation or exercise via a synchronous video-linked appointment.

Adverse events: Any unintended and unfavourable sign, symptom or disease that is associated with the use of an intervention procedure, that may or may not be a direct consequence of the intervention.^14^

### Search strategy

Systematic searches were conducted by one author (RCCB) through four databases from inception to 12 June 2025. The databases included PubMed, Web of Science (Clarivate), Embase (Elsevier) and CINAHL (EBSCOhost). Key search terms included ‘telehealth’, ‘videoconferencing’, ‘telerehabilitation’, ‘exercise’, ‘rehabilitation’, ‘resistance training’, ‘strength training’, ‘aerobic training’, ‘physical therapy’ and ‘physical activity’. Specific search strategies for separate databases are provided in online Supplemental material 1. Recursive and forward searching of reference lists for all included studies and other relevant systematic reviews, including those published in 2024, was completed. Only completed randomised controlled trials were included, and conference abstracts and dissertations were excluded. All authors independently contributed to study selection. Disagreements in screening were resolved through discussion or by a third independent reviewer (RCCB or TGR).

### Eligibility criteria

#### Participants

Included trials were not restricted by age or sex. Trials were included if participants had: (a) a long-lasting chronic condition with persistent effects (e.g. osteoarthritis; chronic stroke) and/or (b) a history of restorative or reconstructive surgery (e.g. coronary artery bypass graft; total hip arthroplasty).

#### Intervention

Trials were included if subjects participated in a physical rehabilitation or exercise intervention delivered via videoconferencing. At least one session in the intervention protocol must have been delivered via videoconferencing by research staff to be included. Sessions must have been supervised by an appropriately qualified health professional. Interventions of any length and follow-up period were included, except those using videoconferencing exclusively for check-ins, non-physical rehabilitation or exercise interventions (e.g. speech pathology, dietetics), or those where digital health tools (e.g. telephone, mobile apps, e-health) were used without videoconferencing sessions.

#### Comparator

Comparator groups must have been in-person exercise therapy supervised by an appropriately qualified health professional for the study to be included. Trials with comparator groups that were not provided an intervention (e.g. continued with current physical activity habits) or that were not in-person exercise therapy (e.g. educational booklets or sessions) were excluded.

#### Outcomes

Trials were included if they reported adverse events for all study groups at study conclusion, or if these data were available upon author contact.

#### Study design

Randomised controlled trials (including pilots and multi-arm trials) were included. All other study designs (e.g. protocols, single arm studies, non-randomised clinical trials) were excluded.

### Data extraction

Data describing trial and participant characteristics, eligibility criteria, exercise or physical rehabilitation protocol and adverse event outcomes were extracted by two independent reviewers (RCCB & RC). Differences between reviewers were mediated through consensus discussion or by a third reviewer (TGR). Where further information was needed, authors were contacted via email by the lead author (RCCB). Adverse events reported were graded for severity using the Common Terminology Criteria for Adverse Events (CTCAE)^14^ as follows: Grade 1 (asymptomatic or mild symptoms), Grade 2 (minimal, local or non-invasive intervention indicated), Grade 3 (severe or medically significant but not immediately life-threatening), Grade 4 (life-threatening consequences) or Grade 5 (death). A consensus approach was used for the classification of adverse event severity. One researcher (RCCB) categorised each adverse event according to the CTCAE system. Each adverse event and associated grading were assessed and verified by a second researcher (TGR). Any cases where there was ambiguity were discussed until consensus was reached. All adverse events included in this review not included in individual publications were obtained via author contact (n = five).

### Study quality and confidence in cumulative estimates

The revised Cochrane Risk of Bias 2 tool^15^ was used for quality appraisal for included trials. Bias was assessed as ‘low’, ‘some concerns’ or ‘high’ across five domains (randomisation process, deviations from intended interventions, missing outcome data, measurement of the outcome, selection of the reported result) by two independent reviewers (RCCB & RC). Disagreements were resolved by discussion or resolution by a third reviewer (JS). The Grading of Recommendations, Assessment, Development and Evaluation^16,17^ was used to assess the certainty of evidence for adverse event outcomes to assess risk of bias, indirectness, inconsistency, imprecision and other factors at the outcome level. Certainty in outcomes were rated as ‘high’, ‘moderate’, ‘low’ or ‘very low’.

### Meta-analysis

The meta-analysis was completed using R version 4.3.3 (R Foundation for Statistical Programming, Vienna, Austria), using the *meta* package version 7.0.0.^18^ The meta-analysis was conducted for between-groups difference in incidence rate of adverse events per person per exercise session, specifically using between-groups incidence rate ratio. Incidence rate was calculated using adverse event counts, sample size and number of prescribed sessions. All adverse events reported in studies were included in the meta-analysis, unless authors stated they were specifically not related to study interventions. Adverse events were sub grouped by severity into minor/moderate (Grade 1 and Grade 2) and major (Grade 3, Grade 4 & Grade 5).^14^ A single three-level meta-analytical model was created, subgrouped by severity, and clustered by study (to minimise issues with double-counting studies with both major and minor/moderate adverse events). Random effects summary estimates were calculated using inverse variance weighting. To account for studies with zero adverse events in both arms, a continuity correction of 0.5 events was applied to studies containing zero events.^19,20^ Heterogeneity was assessed using the I^2^ statistic to account for between-study variability, with values of 0–25%, 26–74% and ≥75% were considered to indicate low, moderate and high heterogeneity, respectively. Due to considerable uncertainty in the effect estimate, sensitivity analyses were not completed.

## Results

**Figure 1.**
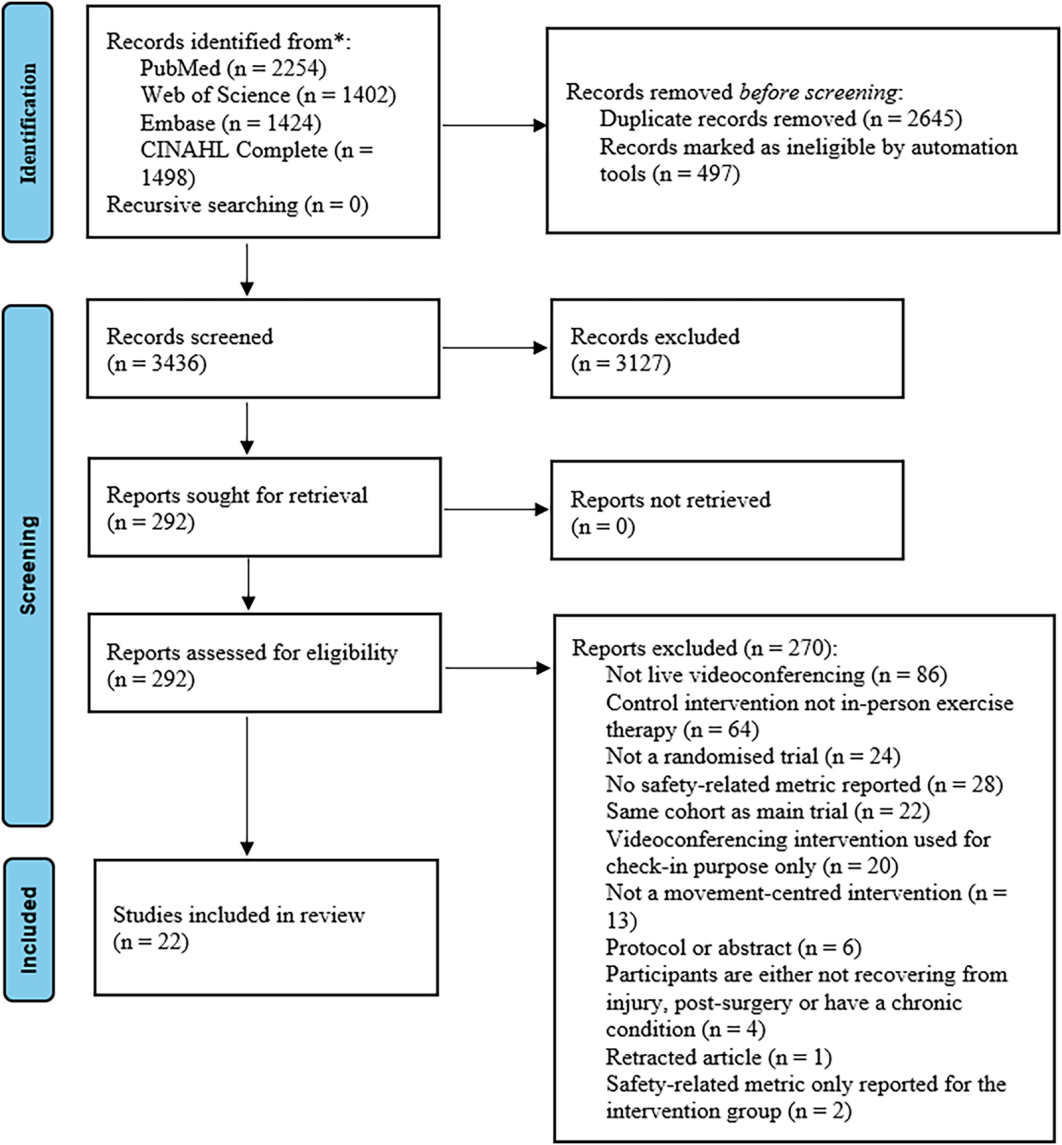
Preferred Reporting Items for Systematic Reviews and Meta-Analysis (PRISMA) flow diagram.

Figure 1 displays that 6578 studies were identified through the initial search. A total of 22 trials were included.

### Trial characteristics

Trial characteristics are described in Table 1. Trials were conducted from 2011 to 2025 in Australia^21–25^ (*n* = 5), United States of America^26–31^ (*n* = 6), China^32,33^ (*n* = 2), Türkiye^34,35^ (*n* = 2) and other countries^36–42^ (*n* = 7). A total of 2082 people participated across all studies. Mean age was 59 ± 8 years. Trials included participants with neurological disorders^27,28,30–33,36,38^ (*n* = 8, 36%), musculoskeletal conditions^22,25,34,35,40,41^ (*n* = 6, 27%), pulmonary diseases^21,37^ (*n* = 2, 9%), cardiac conditions^23,29^ (*n* = 3, 14%), cancer^39^ (*n* = 1, 5%), metabolic disorders^26^ (*n* = 1, 5%), and injury^24^ (*n* = 1, 5%). The most common conditions were stroke^27,30,32,33,38^ and knee arthroplasty,^25,40^ accounting for 538 (26%) of all participants.

**Table 1.**
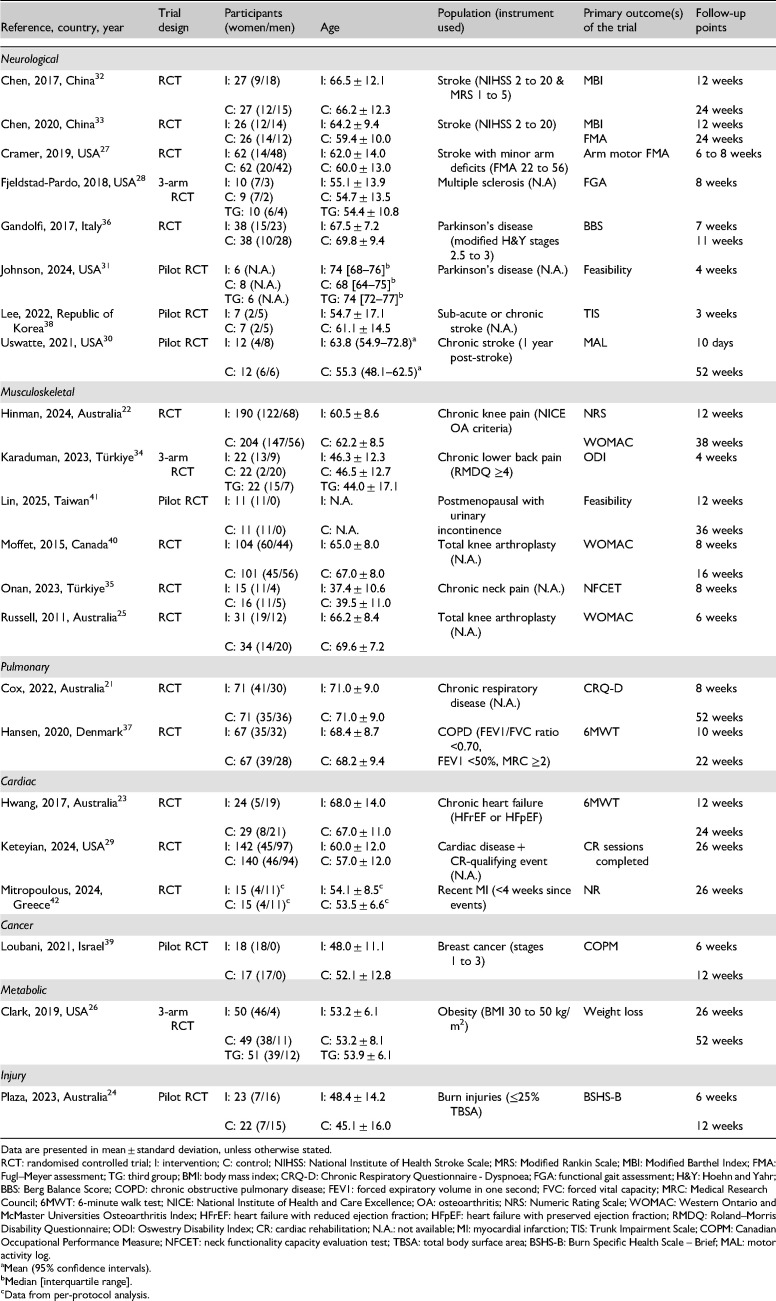
Trial characteristics.

### Intervention characteristics

Details of the videoconferencing interventions are summarised in Table 2. Common types of interventions included resistance exercise^21–27,31,34,35,37,38,40–42^ (*n* = 15), stretching/mobility training^25–27,35,36,40,42^ (*n* = 7), aerobic exercise^21,23,26,29,37,38,42^ (*n* = 7), and balance training^36,38,40,42^ (*n* = 4). Eight trials delivered group-based interventions,^21,23,26,29,35–38^ seven used individual sessions,^22,27,30,32,33,39,40^ one employed a mix of individual and group,^25^ and six did not specify.^24,28,31,34,41,42^ Twelve trials specified the software used for videoconferencing^21–24,27,29,31,36,38–40,42^ and 10 were unspecified or used bespoke systems.^25,26,28,30,32–35,37,41^ Physiotherapists were the most common intervention provider (*n* = 15, 68%). Intervention duration ranged from 10 consecutive weekdays to 52 weeks, with 12 weeks being the most common (*n* = 6, 27%). Participants in most videoconferencing groups were located at home (*n* = 19, 86%). Thirteen trials received additional intervention components, including condition-specific education sessions^22–27,29,37,39–41^ (*n* = 11) and unsupervised exercise programs^21–28,31,40,41^ (*n* = 11). Eleven trials reported session completion,^21–24,26,27,29,30,33,37,41^ with large variability in reporting methods. Mean ± standard deviation was the most common reporting method for session completion (*n* = 4, 36% of trials reporting session completion).

**Table 2.**
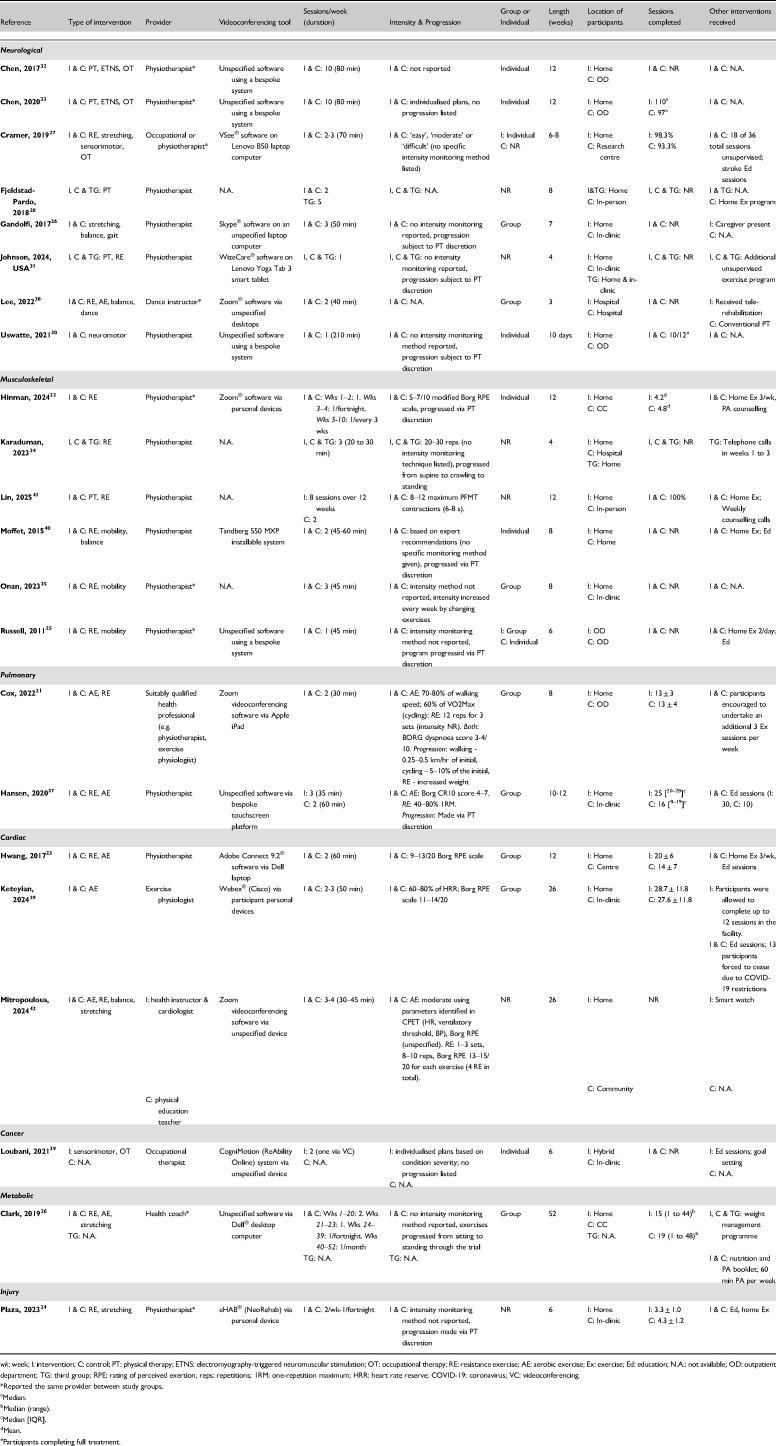
Intervention characteristics.

### Outcome measures

Adverse event data from all included trials are presented in Table 3 and Supplemental material 2. Adverse event assessments were conducted by patient self-report in three trials,^36,39,40^ were clinician-led in ten trials^21–25,29–31,37,41^ and unreported in nine trials.^26–28,32–35,38,42^ Minor/moderate adverse event findings are described (whether they occurred or not) in 21 trials. Ten trials^21–23,26,27,29,31,37,40,41^ described major adverse event findings. Adverse event criteria were defined in nine trials (37%).^21–24,28,29,31,40,41^ Recording methods for adverse events were described in ten trials (42%).^21–24,29,31,36,37,40,41^ The majority of trials (*n* = 12, 55%) reported no adverse events among all participants in the study period.

**Table 3.**
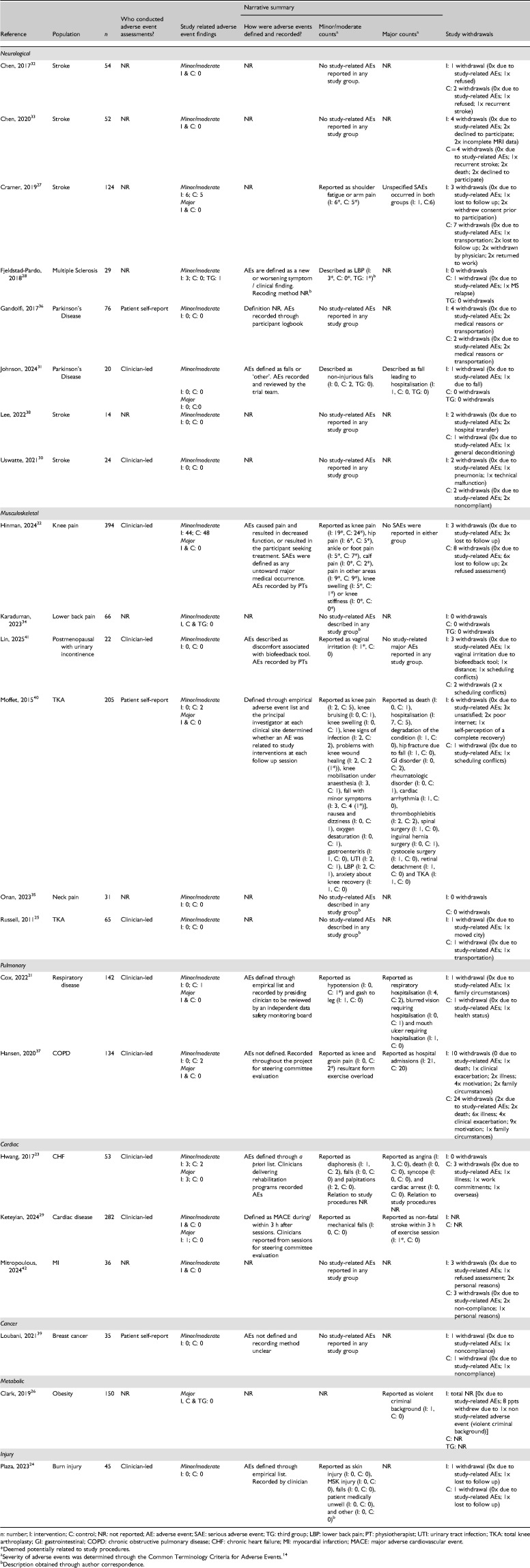
Adverse events.

Study-related adverse events, or adverse events reported with no information regarding study-relation, were reported in nine trials.^21–23,27–29,37,40,41^ Six trials reported adverse events arising from videoconferencing interventions.^22,23,27–29,41^ One trial reported a major adverse event (non-fatal stroke within three hours of exercise) in the videoconferencing group, potentially related to study procedures.^29^ Three cases of angina were reported in one trial in the videoconferencing group, although relation to study procedures was not reported.^23^ The most common study-related adverse events reported were minor/moderate, musculoskeletal in nature (primarily transient pain-related symptoms), present in both study arms and from trials delivering resistance training interventions (*n* = 6, 75% of trials reporting study-related adverse events).^22,27,28,37,40,41^ One study reported two withdrawals in the comparator group as a result of study-related adverse events (knee and groin pain related to exercise overload).^37^

### Risk of bias and certainty in the cumulative effect

**Figure 2.**
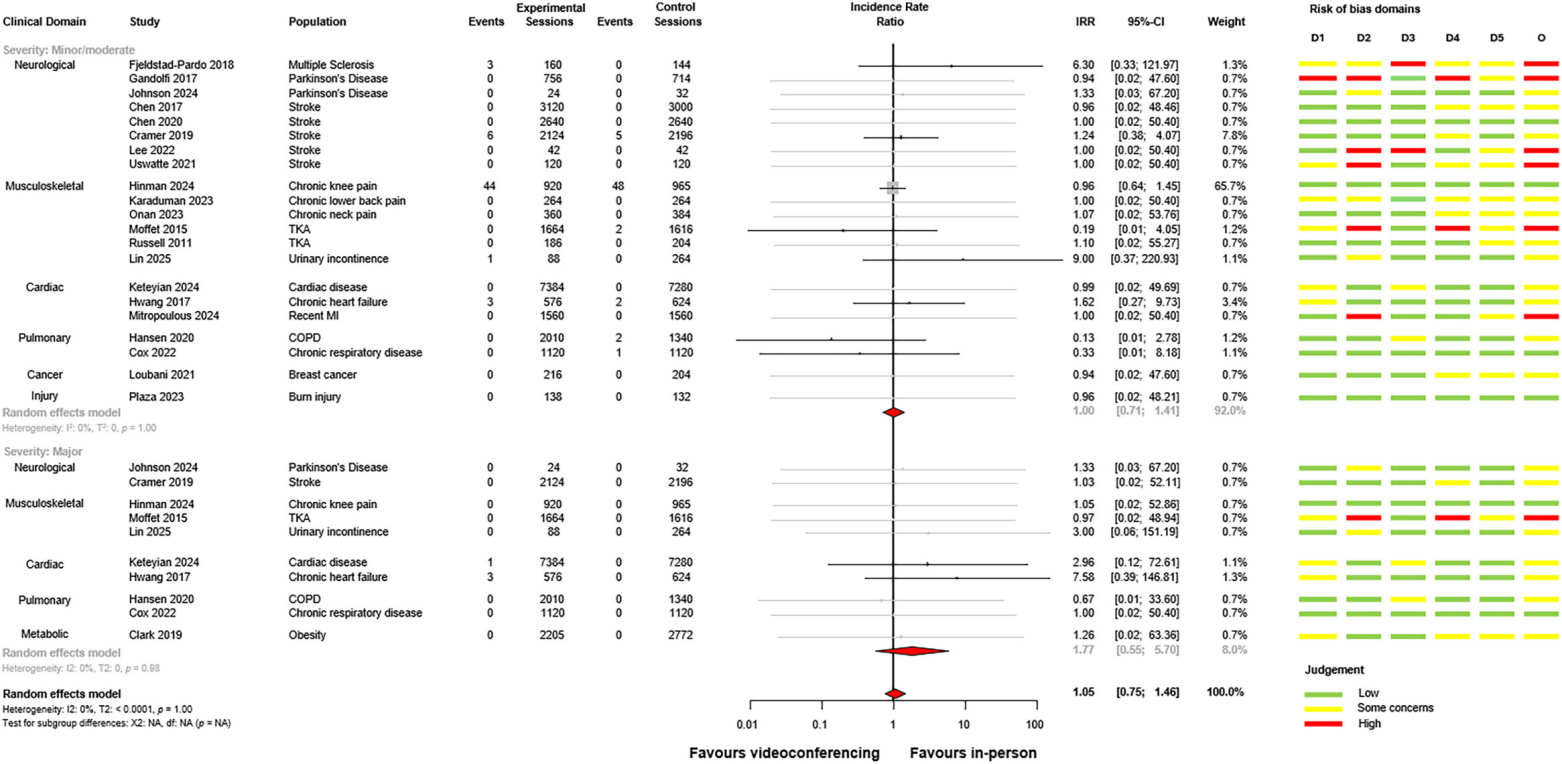
Forest plot of videoconferencing compared to in-person services, sub-grouped by minor/moderate and major adverse events. Studies with no adverse events in either arm are presented in grey on the forest plot.

Risk of bias assessments for individual trials are presented alongside the meta-analysis plot in Figure 2. The overall risk of bias proportions for adverse event outcomes are presented in Supplemental material 3. Most studies had low risk of bias arising from the randomisation process and deviations from intended interventions. Many studies (*n* = 13, 63%) presented bias resulting from the measurement and selection of the reported adverse event results (domains 4 and 5 on the Cochrane Risk of Bias 2 tool). Most trials (*n* = 12, 55%) did not report pre-specified analysis protocols for the monitoring, grading and reporting of adverse events. 18 trials (82%) were rated as ‘some concerns’ or ‘high’ in overall risk of bias for adverse event outcome measures. Certainty in cumulative effect for adverse events was determined to be low via GRADE analysis (Table 4). Adverse event certainty was downgraded for serious risk of bias among studies, and for indirectness of intervention methodology.

**Table 4.** Confidence in cumulative effects – grading of recommendations assessment (GRADE).

Certainty assessment							No. of patients		Effect		Certainty	Importance
№ of studies	Study design	Risk of bias	Inconsistency	Indirectness	Imprecision	Other considerations	Videoconferencing	In-person	Absolute (95% CI)			
Adverse events (follow-up: median 12 weeks)												
22	Randomised trials	Serious^a^	Not serious	Serious^b^	Not serious	None	971	987	**IRR 1.05** (0.75 to 1.46)	⨁⨁◯◯ LOW		CRITICAL

CI: confidence intervals; IRR: incidence rate ratio.

a Eighteen trials (82%) were rated as ‘some concerns’ or ‘high’ using the RoB2 Risk of Bias tool, indicating issues with potential study bias.

b Fifteen trials (68%) included additional intervention strategies for patients beyond videoconferencing (e.g. unsupervised exercise sessions) which may have impacted adverse event outcome measures. No trials reported adverse events alone as a primary outcome measure.

### Meta-analysis

The between-group meta-analysis for videoconferencing versus in-person comparator is presented for adverse events in Figure 2. No statistically significant differences were found between-groups for minor/moderate (incidence rate ratio = 1.00, 95% confidence interval: 0.71 to 1.41, *p* = 0.99, *I*^2^ = 0%) or major (incidence rate ratio = 1.77, 95% confidence interval: 0.55 to 5.70, *p* = 0.98, *I*^2^ = 0%) adverse events. There was considerable uncertainty in the summary estimate for major adverse events, although no significant differences were observed. Overall, it was calculated that one study-related adverse event occurred every 410 videoconferencing sessions, and every 414 in-person sessions. There was no increased incidence of any adverse event for videoconferencing compared to in-person comparators (incidence rate ratio = 1.05, 95% confidence interval: 0.75 to 1.46, *p* = 1.0, *I*^2^ = 0%).

## Discussion

This systematic review and meta-analysis assessed the occurrence of minor/moderate and major adverse events in videoconferencing physical rehabilitation and exercise interventions compared to in-person services. The analyses combined 22 trials including 2082 participants across various population domains. Pooled data suggests that videoconferencing incurs a similar incidence of both minor/moderate and major adverse events as in-person physical rehabilitation or exercise settings. These findings have important ramifications for the promotion of videoconferencing as a safe alternate mode of service delivery where in-person alternatives are not available or preferable. Meta-analysis data suggests that videoconferencing may have a similar number of adverse events as in-person settings across a variety of populations, including high-risk individuals. However, many studies presented risk of bias arising from the measurement, selection and reporting of adverse events. In turn, this decreases confidence and certainty in the findings. While previous literature dictates that videoconferencing is an accepted and effective medium of service delivery,^4,6^ the observations regarding adverse event monitoring and reporting made in this review outline the need for more rigorous methodology for safety-related metrics in future clinical trials.

The findings of this review are largely consistent with previous studies. Videoconferencing interventions have not led to an increased number of adverse events among various clinical groups, including participants eligible for cardiac and pulmonary rehabilitation^10^ and chronic disease generally.^4^ A 2023 rapid review of multidisciplinary telehealth suggests that videoconferencing is not inferior to usual care for hospitalisations or emergency department visits.^11^ Most trials in the current review reported no adverse events in either study arm, and did not report adverse event definitions, recording or reporting methods. Importantly, 28 otherwise eligible trials (10% of all trials excluded at full-text screening phase) were excluded as they did not include adverse events in their study reporting. This inability to verify whether adverse events occurred, or how they were recorded and monitored, makes it difficult to ascertain the true safety of videoconferencing interventions. Future trials should emphasise reporting of adverse events (including relevant reporting methodology) to improve the transparency of safety-related outcomes. While adverse event data was sparse among the included trials, reported study-related minor/moderate adverse events tended to be musculoskeletal in nature, and resulted from trials delivering resistance training. Importantly, occurrence of minor/moderate musculoskeletal adverse events appeared to be similar for both videoconferencing and in-person groups. This increased occurrence following resistance training has been noted in digital health interventions previously,^43,44^ and is noteworthy for clinicians developing exercise prescription over videoconferencing. Notably, none of the included trials monitored or reported intervention adherence for participants after a study-related adverse event. Regardless, while safety is a clinical concern for videoconferencing, there does not appear to be an increased risk of adverse events. This study suggests that there is no evidence to exclude videoconferencing as an option for services based on an increased risk of adverse events alone.

Telehealth literature dictates that videoconferencing is an effective approach to service delivery across a variety of outcomes and different intervention methodologies.^4–6,45,46^ Many trials in this review utilised resistance exercise as a training method. Other forms of exercise therapy (e.g. mobility training, balance exercise and aerobic exercise) can be delivered effectively via videoconferencing.^47–49^ Clinicians are encouraged to base modality selection on individual participant factors, goals and preferences. However, monitoring of session completion only occurred in eleven included trials (50% of total) in this review, and reporting methodology varied widely with no standardised presentation. This observation aligns with previous similar systematic reviews, citing issues with the reporting of session attendance and exercise adherence reporting among trials.^4,6^ While general trends may suggest that videoconferencing leads to similar session completion as in-person settings, future studies must prioritise the reporting of attendance and adherence to understand this observation.

Research trials included in this review typically have been informed by clinical guidelines for the implementation of telehealth interventions, likely leading to the small number of adverse events observed. Therefore, clinicians looking to utilise videoconferencing for physical rehabilitation or exercise therapy should seek to follow established recommendations for the safe conduct of services.^13,50–52^ This includes assessing telehealth readiness on an individual basis with participants, and may consist of the following: (a) assessing clinical status and safety of intervention type using validated tools, (b) determining participant preferences, goals and the suitability of a videoconferencing approach to service delivery based on individual factors,^52^ (c) considering whether the videoconferencing platform selected is compliant with privacy and confidentiality requirements in the location where services are being provided for the use of health information^52^ and (d) considering the participant's physical location and conduct environmental scans to identify potential hazards to safety and other privacy risks.^52^

This review has several important limitations to consider. There was a considerable amount of bias in the measurement and reporting of adverse events among trials, with most trials not reporting detailed analysis protocols. This is likely due to safety metrics not being the primary outcome, and the majority of trials reporting no adverse events. Several trials also relied on participant recollection of adverse events during the study period at end-of-program assessments, potentially leading to recall bias in responses. Most studies reported no adverse events in either treatment arm, leading to large variance in estimated effect sizes for individual studies in the meta-analysis. This led to larger variance in the summary effect for major adverse events, impacting the certainty of our findings. Although the use of a continuity correction to account for zero event studies has been criticised,^19,20^ the use of any alternative method would likely only widen the already broad confidence interval.^53^ Therefore, as these more complex methods were very unlikely to meaningfully alter the conclusions, the simpler continuity correction method was deemed sufficient. Clinical domain subgroup estimates in the meta-analysis were not feasible due to sparse outcome data. While each trial was comparing similar exposures and outcomes and a random-effects meta-analytical model was used, results from multiple clinical domains contributed to the overall effect estimate. Therefore, overall results should be interpreted with caution when applied to specific clinical domains.

Videoconferencing for the provision of physical rehabilitation and exercise services appears to be safe and has a similar number of adverse events as in-person interventions. This systematic review with meta-analysis suggests that there is no evidence to support the notion that videoconferencing should be excluded as an option for engaging with services due to safety risks. However, studies with more robust methodology for the monitoring and reporting of adverse events are needed to improve the certainty of these findings.

**Clinical messages**

- Videoconferencing interventions involving an audio-visual link between health care professionals and patients have been found to be effective and acceptable. However, there is still concern surrounding the safety of these services, particularly from clinicians working with high-risk patients.
- This systematic review with meta-analysis suggests that there is no evidence of a difference in adverse event incidence between videoconferencing and in-person physical rehabilitation or exercise interventions.
- There was a low rate of incidence of adverse events for both videoconferencing and in-person interventions.
- Many studies (55% of all included) did not report pre-specified protocols for the monitoring, grading and reporting of adverse events. More robust trials are needed to verify the findings of this review.

## Supplemental Material

sj-docx-1-cre-10.1177_02692155251361916 - Supplemental material for Safety of videoconferencing for physical rehabilitation and exercise: A systematic review and meta-analysis Supplemental material, sj-docx-1-cre-10.1177_02692155251361916 for Safety of videoconferencing for physical rehabilitation and exercise: A systematic review and meta-analysis by Riley CC Brown, Joshua Simmich, Robert Cuthbert, Megan H Ross, Pablo Molina-Garcia and Trevor G Russell in Clinical Rehabilitation

sj-docx-2-cre-10.1177_02692155251361916 - Supplemental material for Safety of videoconferencing for physical rehabilitation and exercise: A systematic review and meta-analysis Supplemental material, sj-docx-2-cre-10.1177_02692155251361916 for Safety of videoconferencing for physical rehabilitation and exercise: A systematic review and meta-analysis by Riley CC Brown, Joshua Simmich, Robert Cuthbert, Megan H Ross, Pablo Molina-Garcia and Trevor G Russell in Clinical Rehabilitation
