# Supplementary material for: Safety of videoconferencing for physical rehabilitation and exercise: A systematic review and meta-analysis
Source: Clin Rehabil. 2025 Jul 30;39(9):1219–42. doi: 10.1177/02692155251361916 (PMC12340145; doi:10.1177/02692155251361916)
Supplement: sj-docx-1-cre-10.1177_02692155251361916 - Supplemental material for Safety of videoconferencing for physical rehabilitation and exercise: A systematic review and meta-analysis [file sj-docx-1-cre-10.1177_02692155251361916.docx]

**Safety of Videoconferencing for Physical Rehabilitation and Exercise: A Systematic Review and Meta-Analysis**

**Online Supplementary Material**

Contents

[Supplementary Material 1: Database search strategies (PubMed, Web of Science, Embase, Cinahl) 2](#_Toc200525437)

[Supplementary Material 2: Additional intervention and adverse event outcome characteristics 5](#_Toc200525438)

[Supplementary Material 3: Risk of Bias figure 6](#_Toc200525439)

# Supplementary Material 1: Database search strategies (PubMed, Web of Science, Embase, Cinahl)

Searches conducted on 12/06/2025

**PubMed**

| # | Search string | Results |
| --- | --- | --- |
| #1 | "Videoconferencing"[Mesh] OR "Telemedicine"[Mesh] OR "telehealth"[Title/Abstract] OR "tele-health"[Title/Abstract] OR "tele health"[Title/Abstract] OR "telerehabilitation"[Title/Abstract] OR "tele-rehabilitation"[Title/Abstract] OR "tele rehabilitation"[Title/Abstract] OR "videoconferenc*"[Title/Abstract] OR "video conferenc*"[Title/Abstract] OR "video-conferenc*"[Title/Abstract] OR telerehabilitation[Text Word] | 67,931 |
| #2 | "Physical Therapy Modalities"[Mesh] OR "Physical Therapists"[Mesh] OR "Exercise"[Mesh] OR "Exercise Therapy"[Mesh] OR "Cardiac Rehabilitation"[Mesh] OR "Neurological Rehabilitation"[Mesh] OR "exercise*"[Title/Abstract] OR "physical activit*"[Title/Abstract] OR "strength training"[Title/Abstract] OR "resistance training"[Title/Abstract] OR "aerobic training"[Title/Abstract] OR "endurance training"[Title/Abstract] OR "physiotherap*"[Title/Abstract] OR "physio-therap*"[Title/Abstract] OR "physiatrist"[Title/Abstract] OR "rehabilitation*"[Title/Abstract] OR "physical therap*"[Title/Abstract] OR "physical-therap*"[Title/Abstract] OR "manual therap*"[Title/Abstract] OR "manual-therap*"[Title/Abstract] OR "movement"[Title/Abstract] OR "motor"[Title/Abstract] OR rehabilitation[Text Word] OR "tai chi"[Title/Abstract] OR "yoga"[Title/Abstract] OR "balance training"[Title/Abstract] OR "balance retraining"[Title/Abstract] OR "gait training"[Title/Abstract] OR "postural training"[Title/Abstract] OR "strengthening"[Title/Abstract] OR "hydrotherapy"[Title/Abstract] OR "aerobics"[Title/Abstract] OR "McKenzie therapy"[Title/Abstract] OR “functional training”[Title/Abstract] | 1,762,498 |
| #3 | "clinical study"[Publication type] OR "clinical trial"[Text word] OR "randomized controlled trial"[Text word] OR "randomised controlled trial"[Text Word] OR “randomised”[Title/Abstract] OR “randomized”[Title/Abstract] | 1,765,367 |
| #4 | "Systematic Review"[Publication type] OR "Meta-Analysis"[Publication type] OR "Clinical Trial Protocol"[Publication type] OR protocol[Title] | 480,701 |
| #5 | #1 AND #2 AND #3 | 3,108 |
| #6 | #5 NOT #4 | **2,254** |

**Web of Science (Clarivate)**

| # | Search string | Results |
| --- | --- | --- |
| #1 | TS=("telehealth" OR "tele-health" OR "tele health" OR "telerehabilitation" OR "tele-rehabilitation" OR "tele rehabilitation" OR "videoconferenc*" OR "video conferenc*" OR "video-conferenc*") | 42,909 |
| #2 | TS=("exercise*" OR "physical activit*" OR "strength training" OR "resistance training" OR "aerobic training" OR "endurance training" OR "physiotherap*" OR "physio-therap*" OR "physiatrist" OR "rehabilitation*" OR "physical therap*" OR "physical-therap*" OR "manual therap*" OR "manual-therap*" OR "movement" OR "motor" OR “tai chi” OR “yoga” OR “balance training” OR "balance retraining" OR "gait training" OR "postural training" OR "strengthening" OR "hydrotherapy" OR "aerobics" OR "McKenzie therapy" OR “functional training”) | 2,735,345 |
| #3 | ALL=("trial" OR “randomised” OR “randomized”) | 2,148,800 |
| #4 | #1 AND #2 AND #3 | 2,496 |
| #5 | TS=("systematic review" OR “meta analysis” OR “meta-analysis” OR "protocol") OR DT=(“Review” OR “Meeting Abstract”) | 13,888,412 |
| #6 | #4 NOT #5 | **1,402** |

**Embase (Elsevier)**

| # | Search string | Results |
| --- | --- | --- |
| #1 | 'telemedicine'/exp OR 'telehealth':ti,ab,kw OR 'tele-health':ti,ab,kw OR 'tele health':ti,ab,kw OR 'telerehabilitation':ti,ab,kw OR 'tele-rehabilitation':ti,ab,kw OR 'tele rehabilitation':ti,ab,kw OR 'videoconferenc*':ti,ab,kw OR 'video conferenc*':ti,ab,kw OR 'video-conferenc*':ti,ab,kw | 121,610 |
| #2 | 'physiotherapy'/exp OR 'exercise'/exp OR 'kinesiotherapy'/exp OR 'heart rehabilitation'/exp OR 'neurorehabilitation'/exp OR 'exercise*':ti,ab,kw OR 'physical activit*':ti,ab,kw OR 'strength training':ti,ab,kw OR 'resistance training':ti,ab,kw OR 'aerobic training':ti,ab,kw OR 'endurance training':ti,ab,kw OR 'physiotherap*':ti,ab,kw OR 'physio-therap*':ti,ab,kw OR 'physiatrist':ti,ab,kw OR 'rehabilitation*':ti,ab,kw OR 'physical therap*':ti,ab,kw OR 'physical-therap*':ti,ab,kw OR 'manual therap*':ti,ab,kw OR 'manual-therap*':ti,ab,kw OR 'movement':ti,ab,kw OR 'motor':ti,ab,kw OR ‘tai chi’:ti,ab,kw OR ‘yoga’:ti,ab,kw OR ‘balance training’:ti,ab,kw OR ‘balance retraining’:ti,ab,kw OR ‘gait training’:ti,ab,kw OR ‘postural training’:ti,ab,kw OR ‘strengthening’:ti,ab,kw OR ‘hydrotherapy’:ti,ab,kw OR ‘aerobics’:ti,ab,kw OR ‘McKenzie therapy’:ti,ab,kw OR ‘functional training’:ti,ab,kw | 2,227,555 |
| #3 | 'clinical trial'/exp AND ‘random*’:ti,ab,kw AND ‘article’:it | 577,401 |
| #4 | 'systematic review'/exp OR 'systematic review':ti OR 'meta-analysis':ti OR 'meta analysis':ti OR 'protocol':ti | 776,916 |
| #5 | #1 AND #2 AND #3 | 1,785 |
| #6 | #5 NOT #4 | **1,424** |

**Cinahl (EBSCOhost)**

| # | Search string | Results |
| --- | --- | --- |
| #1 | MM "Telehealth" OR MH "Telemedicine+" OR MH "Videoconferencing+" OR TI ( "telehealth" OR "tele-health" OR "tele health" OR "telerehabilitation" OR "tele-rehabilitation" OR "tele rehabilitation" OR "videoconferenc*" OR "video conferenc*" OR "video-conferenc*" ) OR AB ( "telehealth" OR "tele-health" OR "tele health" OR "telerehabilitation" OR "tele-rehabilitation" OR "tele rehabilitation" OR "videoconferenc*" OR "video conferenc*" OR "video-conferenc*" ) | 52,554 |
| #2 | MM "Rehabilitation" OR MH "Physical Therapy+" OR MH "Rehabilitation, Cardiac+" OR MM "Rehabilitation, Vocational" OR TI ( "exercise*" OR "physical activit*" OR "strength training" OR "resistance training" OR "aerobic training" OR "endurance training" OR "physiotherap*" OR "physio-therap*" OR "physiatrist" OR "rehabilitation*" OR "physical therap*" OR "physical-therap*" OR "manual therap*" OR "manual-therap*" OR "movement" OR "motor" OR “tai chi” OR “yoga” OR “balance training” OR "balance retraining" OR "gait training" OR "postural training" OR "strengthening" OR "hydrotherapy" OR "aerobics" OR "McKenzie therapy" OR “functional training” ) OR AB ( "exercise*" OR "physical activit*" OR "strength training" OR "resistance training" OR "aerobic training" OR "endurance training" OR "physiotherap*" OR "physio-therap*" OR "physiatrist" OR "rehabilitation*" OR "physical therap*" OR "physical-therap*" OR "manual therap*" OR "manual-therap*" OR "movement" OR "motor" OR “tai chi” OR “yoga” OR “balance training” OR "balance retraining" OR "gait training" OR "postural training" OR "strengthening" OR "hydrotherapy" OR "aerobics" OR "McKenzie therapy" OR “functional training” ) | 578,315 |
| #3 | PT “clinical trial” OR TX “clinical trial” OR TX “randomized” OR TX “randomised” | 725,070 |
| #4 | #1 AND #2 AND #3 | 1,833 |
| #5 | PT “systematic review” OR PT “Meta-Analysis” OR TI “Protocol” | 210,482 |
| #6 | #4 NOT #5 | **1,498** |

# Supplementary Material 2: Additional intervention and adverse event outcome characteristics

| Reference | Did the trial employ the same providers between intervention and control? | Whether dropouts or adherence issues were tracked in relation to adverse event occurrences. | Whether there were differences in adherence between those who experienced adverse events and those who didn’t |
| --- | --- | --- | --- |
| *Neurological* |  |  |  |
| Chen 2017 | Same physiotherapists | I & C: NR* | I & C: NR* |
| Chen 2020 | Same physiotherapists | I & C: NR* | I & C: NR* |
| Cramer 2019 | Same occupational therapists or physiotherapists | I & C: NR | I & C: NR |
| Fjeldstad-Pardo 2018 | NR | I, C & TG: NR | I, C & TG: NR |
| Gandolfi 2017 | Different physiotherapists | I & C: NR* | I & C: NR* |
| Johnson 2024 | NR | I & C: Yes | I: One ppt withdrew after a non-study-related fall leading to hospitalisation  C: NR |
| Lee 2022 | Same dance instructors | I & C: NR* | I & C: NR* |
| Uswatte 2021 | NR | I & C: NR* | I & C: NR* |
| *Musculoskeletal* |  |  |  |
| Hinman 2024 | Same physiotherapists | I & C: Yes | I & C: No differences |
| Karaduman 2023 | NR | I, C & TG: NR* | I, C & TG: NR* |
| Lin 2025 | NR | I & C: Yes | I: One ppt withdrew after a study-related adverse event (irritation)  C: NR |
| Moffet 2015 | Different physiotherapists | I & C: Yes | I & C: No differences |
| Onan 2023 | Same physiotherapists | I & C: NR* | I & C: NR* |
| Russell 2011 | Same physiotherapists | I & C: NR* | I & C: NR* |
| *Pulmonary* |  |  |  |
| Cox 2022 | Different suitably qualified health professionals | I & C: Yes | I & C: No differences |
| Hansen 2020 | NR | I & C: Yes | I & C: NR (beyond withdrawal information) |
| *Cardiac* |  |  |  |
| Hwang 2017 | NR | I & C: NR | I & C: NR |
| Keteyian 2024 | NR | I & C: NR | I & C: NR |
| Mitropoulous 2024 | Different instructors | I & C: NR* | I & C: NR* |
| *Cancer* |  |  |  |
| Loubani 2021 | NR | I & C: NR* | I & C: NR* |
| *Metabolic* |  |  |  |
| Clark 2019 | Same health coaches | I=Yes  C, TG: NR* | I: 8 ppts withdrew and did not receive intervention after one non study-related adverse event experienced  C, TG: NR* |
| *Injury* |  |  |  |
| Plaza 2023 | Same physiotherapists | I & C: NR* | I & C: NR* |

*No adverse events reported. Abbreviations: *C*, Control; *I*, Intervention; *TG*, Third group; *NR*, Not reported.

# Supplementary Material 3: Risk of Bias figure

Supplementary material 3 Proportions of risk of bias for all studies for adverse event outcomes
